# Clays and Wound Healing

**DOI:** 10.3390/ma17071691

**Published:** 2024-04-07

**Authors:** Guangjian Tian, Zhou Wang, Zongwang Huang, Zuyan Xie, Lu Xia, Yi Zhang

**Affiliations:** 1 Department of Inorganic Materials, School of Minerals Processing and Bioengineering, Central South University, Changsha 410083, China; 215612081@csu.edu.cn (G.T.); zhouwang@csu.edu.cn (Z.W.); zwanghuang@csu.edu.cn (Z.H.); 2 Center for Medical Genetics & Hunan Key Laboratory of Medical Genetics, School of Life Sciences, Central South University, Changsha 410078, China; xiezuyan@sklmg.edu.cn

**Keywords:** clay, aluminosilicate, hemostasis mechanism, ionic effect, hemostatic material

## Abstract

Aluminosilicates, such as montmorillonite, kaolinite, halloysite, and diatomite, have a uniform bidimensional structure, a high surface-to-volume ratio, inherent stiffness, a dual charge distribution, chemical inertness, biocompatibility, abundant active groups on the surface, such as silanol (Si-OH) and/or aluminol (Al-OH) groups. These compounds are on the list of U.S. Food and Drug Administration-approved active compounds and excipients and are used for various medicinal products, such as wound healing agents, antidiarrheals, and cosmetics. This review summarizes the wound healing mechanisms related to the material characteristics and the chemical components. Numerous wound dressings with different active components and multiple forms have been studied. Then, medicinal mineral resources for use in hemostatic materials can be developed.

## 1. Introduction

In various societal accidents, numerous wounded people die due to massive hemorrhage and ineffective hemostasis [1,2,3]. The development of hemostatic materials with various active components and in different forms to achieve more efficient hemostasis in wound healing has continued to increase during long-term studies [4,5,6,7]. These commercial hemostatic materials include kaolinite, which is used in combat gauze from Z-Medica Corp. (Wallingford, CT, USA); zeolite, which is used in QuikClot from Z-Medica Corp.; montmorillonite, which is used in WoundStat from TraumaCure Inc. (Bethesda, MD, USA); mesoporous silicon and chitosan, which are used in TraumaStat from Ore-Medix Inc. (Salem, OR, USA); chitosan, which is used in Celox from MedTrade Inc. (Houston, TX, USA) and ChitoGauze Pro from HemCon Inc. (Portland, OR, USA); and cellulose, which is used in Traumastem from Bioster Inc. (Stillwater, MN, USA) [8]. These hemostatic materials act by absorbing water from the blood and concentrating the blood components, activating platelets and blood coagulation cascade reactions, and providing a physical barrier at the hemorrhagic site. In addition, multiple forms, including particles, hydrogels, nanofibers, and sponges, also influence their functions and applications.

Medicinal mineral resources with less market share should be noted, such as cellulose, starch, collagen, chitosan, fibrinogen, etc., because these medicinal mineral resources are distinctive components in traditional Chinese medicine. Their characteristics, including chemical compositions, internal structures, and trace elements, will influence medicinal methods, medicinal efficacies, and remedy-based results. There are more than 80 kinds of medicinal mineral resources, including metals and nonmetals in single-element minerals and compound minerals, which are substances, excipients, or additives in antibacterial, hemostasis, wound healing, and other therapeutic drug applications [9,10]. Hemostatic minerals are distinctive and indispensable components in traditional Chinese medicinal mineral resources. They are present in compound minerals, such as stalactite, calcite, limonitum, ochre, kaolinite, montmorillonite, and zeolite, more often than in single-element minerals.

**Figure 1:**
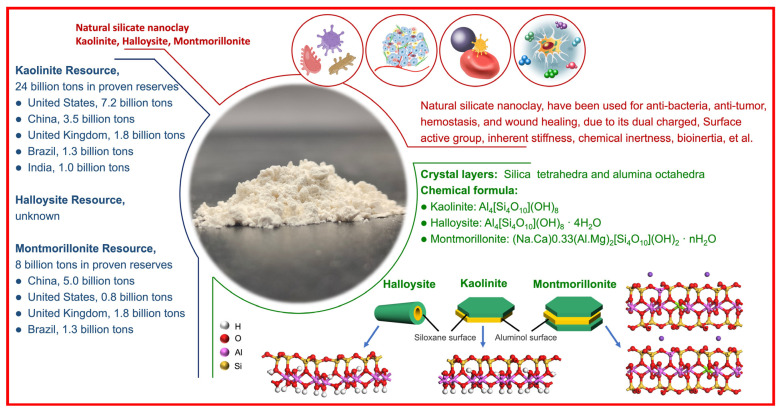
An overview diagram for kaolinite, halloysite, and montmorillonite.

Natural aluminosilicate clay minerals refer to those materials containing Si-O-Al linkages, sometimes accompanied by variable amounts of iron (Fe), magnesium (Mg), titanium (Ti), alkali metals, alkaline earth, and other cations. Natural aluminosilicate clays, such as montmorillonite, kaolinite, halloysite, and diatomite, are used as multinational Food and Drug Administration-approved excipients or additives in various biomedical applications owing to their characteristics, such as natural shape, large specific surface area, high surface-to-volume ratio, dual-charge distribution, active groups, inherent stiffness, chemical inertness, high adsorption and swelling capacities, and biocompatibility [11,12,13,14,15]. Kaolinite, with the theoretical chemical formula of Al_2_Si_2_O_5_(OH)_4_, consists of one Al-octahedral sheet and one Si-tetrahedral sheet. The active groups include Al-OH groups on the outer surface and Si-OH groups or Al-OH groups on the end face [16,17]. Halloysite, with the theoretical chemical formula Al_2_Si_2_O_5_(OH)_4_·nH_2_O, consists of one Si-tetrahedral sheet-based outer surface and one Al-octahedral sheet-based inner surface. The active groups include Al-OH groups on the inner surface, Si-OH groups on the outer surface, and Al-OH groups or Si-OH groups on the end faces. The dual-charge distribution (negative charges on the outer surface and opposite charges on the inner surface) results from the different groups on the inner surface and outer surface [18,19]. Montmorillonite, with the theoretical chemical formula (M^+^_x+y_·nH_2_O){(A1_2−x_Mg_x_)[Si_4_O_10_](OH)_2_}, consists of one Al-octahedral sheet and two Si-tetrahedral sheets, where M refers to an exchangeable cation. According to the literature, as of August 2020, there were 222 commercial clay-based commodities with nanotechnologies on the global market, involving 25 countries, 125 companies, and 70 items. The United States ranked first in total, with 66 commodities, 36 companies, and 28 items, and food items were ranked first. China ranked second in total, with 39 commodities, 21 companies, and 16 items, and medicine items were ranked first (Figure 1) [20].

In this review, the hemostasis mechanism related to the basic characteristics and chemical composition are studied, numerous wound dressings with different active components and multiple forms are described, and then an outlook for the future is presented.

## 2. Hemostatic Mechanism

Hemostasis is the first stage in wound healing and immediately starts to reduce blood loss at the wound site in the first minutes. Hemostatic materials are more effective in controlling hemorrhage and even in accelerating wound healing abilities. Therefore, the hemostatic material and its hemostatic biological mechanisms should be summarized in a continuous manner that could be a valuable reference in clinical therapeutics. The hemostatic mechanism for clays is related to their internal structures (one-dimensional or two-dimensional), characteristics (surface roughness, the surface/end charge, and wettable surface), main chemical compositions (silicon, Si; aluminum, Al), trace elements (calcium, Ca; zinc, Zn; copper, Cu; et al.), etc. The nano-bio interactions and hemostatic effects following treatment with clays at the cellular, molecular, and in vivo levels are being revealed.

### 2.1. Hemostatic Mechanism Related to Material Characteristics

**Figure 2:**
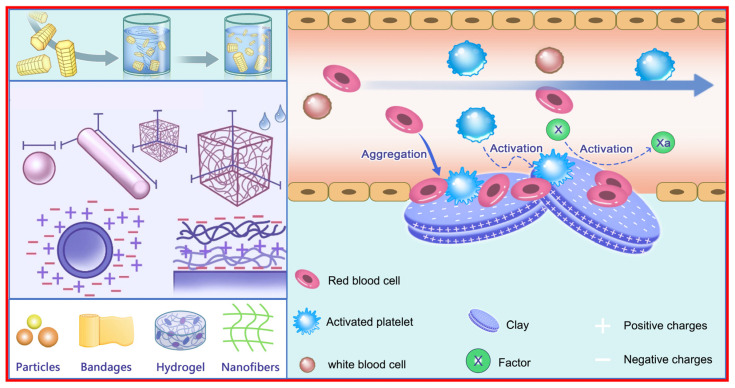
An overview of the hemostatic mechanism related to material characteristics. Reprinted with permission from ref. [4]. 2023, Elsevier.

In this section, the hemostatic mechanism related to the surface roughness, the surface/end charge, and the wettable surface are discussed (Figure 2).

#### 2.1.1. Surface Roughness

Clay surface morphologies, including surface roughness, surface curvature, and surface texture, could affect various protein adsorption properties, including the total adsorption amount, adsorption thickness, competitive adsorption, and conformational changes [21,22].

Clays first adsorb plasma proteins in seconds after contact with the blood due to the concentration and diffusion rate in blood. Blood coagulation-related plasma proteins include albumin, γ-globulin, and fibrinogen. Albumin adsorption helps to inhibit blood coagulation on the clay surface. Fibrinogen adsorption and its conformational changes activate blood coagulation factors and platelets, and then thrombi form.

Research has shown that a higher surface roughness results in a larger exposed area in blood and easier clotting; i.e., surface roughness correlates with clotting [22,23]. Notably, clay surface modification can enhance surface smoothness, which is beneficial for reducing thrombosis.

#### 2.1.2. Surface Charge

Clay surface charges result from the internal structure, chemical composition, and exchangeable ions of these materials. Clays often have a dual charge distribution and a net negative charge in aqueous suspensions.

Most blood components have a negative charge and can be adsorbed onto clay surfaces with opposite charges through electrostatic attraction, causing coagulation and thrombosis. Notably, a clay surface with a negative charge can activate coagulation factor XII and subsequently cause intrinsic coagulation, even without the adsorption of blood components and cell adhesion [24,25].

Research has shown that clay surfaces with different charge distributions and the amount of charge in units influence blood coagulation. Surface modification with the charged groups to obtain a suitable charge distribution range on the clay surface could facilitate blood coagulation.

#### 2.1.3. Wettable Surface and Other Factors

Clay surface groups, including -OH, -COOH, and even -NH_2_, could facilitate the formation of a water-wettable region used in protein adsorption [26]. In addition, clays with higher surface free energy result in larger clay-blood component interactions, which could facilitate protein adsorption and blood coagulation. Notably, clay surfaces with hydrophobic groups could not adsorb those proteins efficiently because of the large interfacial free energies, so blood coagulation activation was needed; that is, the adhesion characteristics between the clay surface and the blood components were much smaller than the cohesion characteristics between the blood components within the blood.

### 2.2. Hemostatic Mechanism Related to Chemical Components

**Figure 3:**
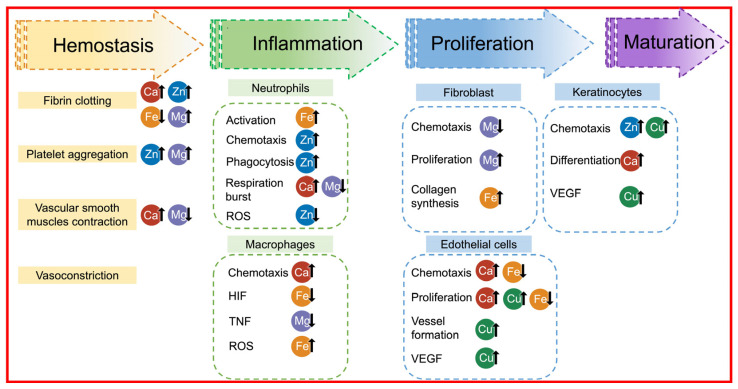
An overview of the hemostatic mechanism related to chemical components. HIF: hypoxia-inducible factor; TNF: tumor necrosis factor; VEGF: vascular endothelial growth factor; ROS: reactive oxygen species. Reprinted with permission from ref. [27]. 2019, Elsevier.

Aluminosilicate clays mainly consist of SiO_2_ and Al_2_O_3_ and contain certain amounts of Fe_2_O_3_ and MgO, as well as small amounts of K_2_O, Na_2_O, and CaO. Skin’s surface environment is weak acidic, and weak acidic environments have been shown to aid wound healing. Metal cations could come out from these metal oxides in weak acidic conditions and even in neutral solutions. Besides, clay surfaces with negative charges tend to attract the opposite cations. In particular, Ca^2+^, Zn^2+^, Fe^2+^, and Mg^2+^ can facilitate blood coagulation (Figure 3) [27,28].

#### 2.2.1. Calcium Ions

In intrinsic coagulation pathways, calcium ions can assist in factor IX (FIX) activation and even activate factor X (FX) through combination with activated (FIX). In extrinsic coagulation pathways, calcium ions can accelerate the binding of exposed tissue factor (TF) and factor VII (FVII), which activate FX. In common pathways, calcium ions can facilitate prothrombin transformation into thrombin, assist with phospholipids, and activate FX, which can accelerate fibrinogen conversion into fibrin monomers. In addition, calcium ions can enhance platelet aggregation in platelet-rich plasma and assist in factor XIII (FXIII) activation, which converts soluble fibrin monomers into stable fibrin multimers [29].

The epidermis is composed of the basal layer, spinous layer, granular layer, transparent layer, and stratum corneum. Keratinocytes are the main constituent cells of the epidermis and are tightly connected by intercellular bridges, forming a barrier. The calcium ion concentration affects keratinocyte proliferation, keratinocyte differentiation, keratinocyte-to-keratinocyte junctions, and keratinocyte barrier function in the epidermis [30,31,32]. The calcium ion concentration inside and outside the keratinocytes from the basal cell layer to the granular layer increases from a low level to a high level, while the calcium ion concentration in the stratum corneum is low. This calcium ion concentration gradient causes the different layers of the epidermis to undergo different degrees of differentiation. Notably, the calcium ion concentration gradient from the inside to the outside of keratinocytes is maintained by the active transport of the cell membrane, endoplasmic reticulum, and inner mitochondrial membrane. The molecular biological mechanism by which calcium ions regulate the division and differentiation of keratinocytes involves increasing the intracellular calcium ion concentration, linking calmodulin with intracellular calcium ions, activating various intracellular enzymes by calmodulin, and regulating the division and differentiation of keratinocytes. Low extracellular calcium concentrations promote keratinocyte proliferation, and high extracellular calcium concentrations promote keratinocyte differentiation and intercellular adhesion.

**Figure 4:**
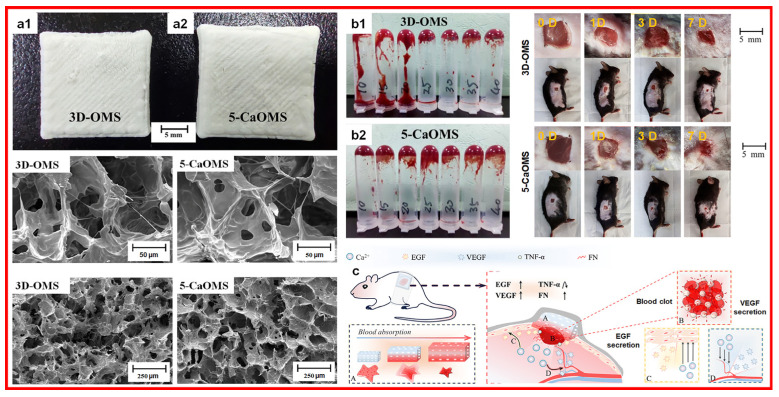
Calcium ions incorporated microporous hydrogel with its (**a1**,**a2**) photos and SEM images, (**b1**,**b2**) in vitro and in vivo hemostatic evaluation, and (**c**) wound healing mechanism. 3D-OMS: microporous oxidized maize starch; Ca-OMS: Ca^2+^ based oxidized maize starch. Reprinted with permission from ref. [36]. 2023, RSC.

Various studies have reported the use of calcium-based biomaterials in wound healing [33]. Calcium-crosslinked alginates have been used for acute and chronic wound healing because calcium ions can assist the clotting cascade [34]. Moreover, calcium carbonate nanoparticles (topical injection or intravenous injection) and calcium phosphate particles (topical injection or topical dressing) have promoted calcium ion-induced wound healing. During wound healing, calcium-containing bioglass has been shown to promote the proliferation, migration, and protein and growth factor expression of endothelial cells and fibroblasts [35]. Calcium ions incorporated microporous hydrogel (illustrated in Figure 4) that could accelerate wound healing through the blood or tissue fluid absorbing on the wound surface due to the hydrogel’s characteristics and activating the coagulation cascade with calcium ions released [36].

#### 2.2.2. Zinc Ions

Zinc ions are involved in more than 300 enzymes and several transcription factors. During wound healing, zinc ions are involved in epithelial tissue differentiation, metallothionein transport, and storage, reducing UV-induced cell and gene damage and improving the tolerance of skin fibroblasts to oxidative stress [37].

Zinc ion levels in plasma have a significant effect on hemostasis [38,39]. Zinc ions can regulate high-molecular-weight kininogen (HMWK) and factor XII (FXII) to achieve negatively charged surface binding to potentiate the intravascular clotting process. In particular, the combination of HMWK and vascular endothelial cells is important for the activation of FIX and factor XI (FXI) [40,41]. In addition, zinc ions can protect calcium ion channels through chelation, ensure the production of calcium-dependent protein kinases during platelet activation, and then facilitate platelet activation [34]. Clinical research has shown that low zinc intake causes poor platelet aggregation and an increased bleeding tendency in adult males, and this condition can be remedied through zinc supplementation.

Zinc is an essential trace element for maintaining the human immune system and immune cell metabolism. Zinc ions are important for the directed proliferation, apoptosis, and intracellular signaling of immune cells [42,43,44]. Low levels of zinc ions can reduce neutrophil chemotaxis and phagocytosis. Zinc ions are directly involved in protein synthesis, and low zinc concentrations can also affect protein structures and charge states. Zinc ions bind to different amino acid ligands and have different functions, such as maintaining protein structure, redox balance, and cell cycling. For example, zinc ions can affect the ion channel conformation through binding to histidine (His), cysteine (Cys), aspartic acid (Asp), and glutamic acid (Glu) residues. Ion channels are important in various immune cell signaling pathways, especially in T cells and B cells. This explains the indirect effects of zinc ions on intracellular signaling and immune responses [45].

Zinc ions play an important role in the wound-healing process by supporting tissue growth and repair [46,47]. Zinc-dependent matrix metalloproteases can degrade almost all components in the extracellular matrix. These endopeptidases are called matrix metalloproteinases because certain metal ions are required as active sites. These enzymes can originate from several different cells in the wound, such as keratinocytes, fibroblasts, macrophages, endothelial cells, mast cells, and eosinophils. Zinc ion-dependent endopeptidases play important roles in cell proliferation, migration, differentiation, angiogenesis, apoptosis, and host defense [48]. In addition, zinc is similar to copper and manganese and can enhance autologous debridement and keratinocyte migration during wound healing.

**Figure 5:**
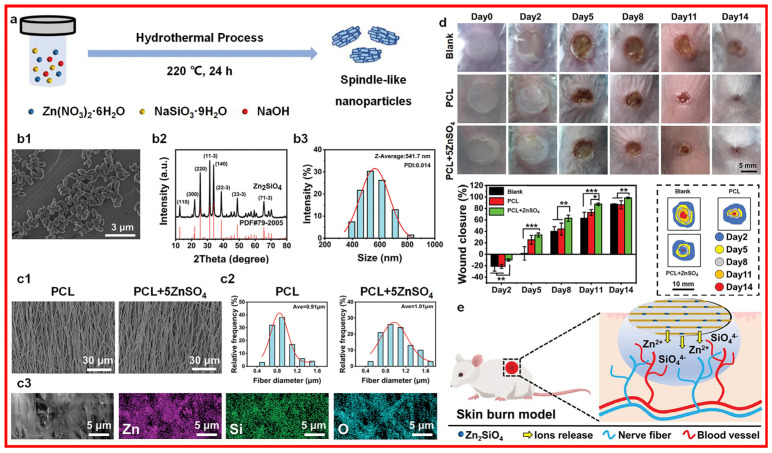
Zinc ions incorporated scaffold with its (**a**) fabrication illustration, (**b1**–**b3**,**c1**–**c3**) materials characterization ((**b1**): SEM; (**b2**): XRD: (**b3**): DSL; (**c1**): SEM; (**c2**): DSL; (**c3**): element mappings), (**d**) in vivo skin burn wound healing evaluation, and (**e**) wound healing mechanism. PCL: poly(ε-caprolactone). * *p* < 0.05, ** *p* < 0.01, *** *p* < 0.001. Reprinted with permission from ref. [49]. 2023, Wiley.

Research has shown that zinc oxide nanoparticles can produce reactive oxygen species (ROS). They can also facilitate cell migration and adhesion and even accelerate the wound-healing process by triggering growth factor-mediated pathways. With the generation of ROS, zinc oxide nanoparticles inhibit the expression levels of superoxide dismutase and glutathione peroxidase genes in human keratinocytes and induce oxidative stress and apoptosis in the cell membrane. Furthermore, the higher zinc oxide nanoparticle concentrations are associated with mitochondrial dysfunction in keratinocytes, releasing lactate dehydrogenase. Zinc ions incorporated scaffold (illustrated in Figure 5) could accelerate the innervated and vascularized skin burn wound healing through the sustained released zinc ions that could enhance the angiogenic abilities and neurogenic activities in vitro [49].

#### 2.2.3. Iron Ions

Iron is an indispensable trace element in humans, and its absorption occurs in the duodenum. Most iron exists in red blood cells in the form of hemoglobin, and the free Fe^3+^ in the plasma can combine with proteins or enzymes to maintain the normal function of the human body. Hepcidin is a peptide hormone that regulates iron homeostasis during iron metabolism, inhibits iron absorption via intestinal mucosal epithelial cells, and regulates the absorption, transport, and utilization of iron in the body.

Thrombin can activate FXIII to generate FXIIIa, convert soluble fibrinogen in plasma into insoluble fibrin monomers, and then the insoluble fibrin monomers interweave into a network to form firm fibrin polymers to achieve rapid hemostasis. Thus, iron ions have a certain influence on fibrin clot formation because iron ions easily combine with protein, Fe^3+^ can react with hemoglobin in blood and aggregate into thrombi blocked in blood vessels, and free Fe^3+^ modifies fibrinogen molecules for resistant fibrinolysis and stimulates the coagulation cascade reaction, and these phenomena have roles in thrombosis [50]. Studies have shown that iron oxide nanoparticles can affect thrombin clotting activities [51]. A lower iron content can cause increased reactive thrombocytosis and then lead to thrombosis. Excess ferrous ions can cause an increase in hydroxyl radicals and then accelerate thrombosis.

Ferrous Fe ions is a prolyl hydroxylase (heme iron(II)-independent dioxygenase) cofactor that could participate in the regulation of hypoxia inducible factor-1α (HIF-1α) hydroxylation during hypoxia. A lower iron ion content results in HIF-1α accumulation due to its stabilization. The increased levels of HIF-1α and vascular endothelial growth factor (VEGF) can be beneficial for angiogenesis and even in wound healing. However, HIF-1α accumulation affects the expression of the proinflammatory cytokine macrophage migration inhibitory factor, which could cause inflammation.

**Figure 6:**
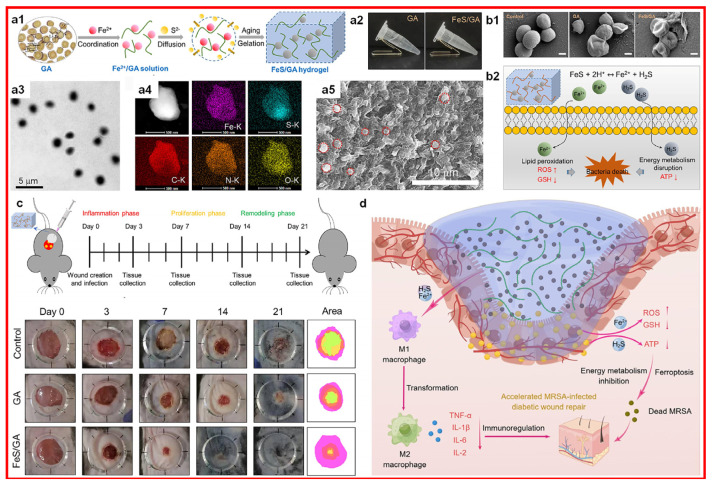
Iron ion incorporated hydrogel with its (**a1**–**a5**) fabrication illustration and materials characterization ((**a1**): fabrication illutration; (**a2**): photos; (**a3**): FeS’s TEM; (**a4**): FeS’s elemental mapping; (**a5**): FeS/GA hydrogel’s SEM, with red circles indicating FeS), (**b1**,**b2**) antibacterial characterization and mechanism, (**c**) in vivo infected diabetic wound healing evaluation, and (**d**) wound healing mechanism. GA: glycyrrhizic acid. Scale bars in b1 is 400 nm. Reprinted with permission from ref. [53]. 2023, Elsevier.

Research has shown that Fe doping and NIR laser irradiation contribute to fibroblast proliferation, neovascularization, and collagen deposition, thus enabling the iron-doped carbon dots-mediated healing of bacteria-infected wounds [52]. Fe doping endows carbon dots with photo-enhanced peroxidase-like activity, which leads to the generation of heat and ROS to kill gram-positive and gram-negative bacteria. Iron ion incorporated hydrogel (illustrated in Figure 6) that could accelerate the infected diabetic wound healing through the released ferrous Fe ions that could induce bacterial death [53].

#### 2.2.4. Copper Ions

Copper is an indispensable trace element in humans, and its absorption occurs in the small intestine and in small amounts in the stomach [54]. Copper ions have an important role in metabolism because they are important components in metalloenzymes, such as ceruloplasmin, cytochrome C oxidase, copper-zinc superoxide dismutase, tyrosinase, lysyl oxidase, and dopamine-beta-hydroxylase. Therefore, ceruloplasmin could activate ferroxidase and amine chlorinate, which can regulate iron absorption and transport. In addition, copper is a component of coagulation factor V (FV) and metallothionein.

Copper protein plays different roles in biological electron transport and oxygen transport due to the interconversion of Cu^1+^ and Cu^2+^ [55,56]. Cytochrome C oxidase plays a role in oxygen reduction and energy generation. Copper-zinc superoxide dismutase converts superoxide into oxygen molecules and hydrogen peroxide for antioxidant defense. Tyrosinase converts tyrosine into melanin and is involved in collagen synthesis and elastin synthesis in bone and connective tissue. Dopamine beta-hydroxylase has a role in the conversion of dopamine to norepinephrine.

Copper plays an important role in inducing angiogenesis by acting on various angiogenic factors, such as VEGF, angiopoietin (ANG), platelet-derived growth factor (PDGF), fibroblast growth factor 1 (FGF1), fibroblast growth factor 2 (FGF2), and interleukin 1 (IL-1). In addition, copper can affect endothelial cells by binding angiogenin and tripeptide glycyl-L-histidyl-L-lysine (GHK), which then play a role in promoting/modulating dermal wound healing [57,58,59].

**Figure 7:**
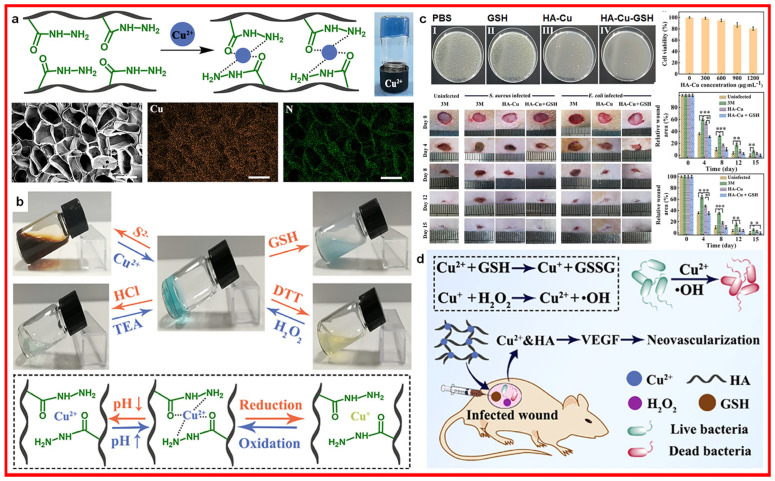
Copper ion incorporated hydrogel with its (**a**) fabrication illustration and materials characterization, (**b**) schematic illustration with triple stimuli-responsiveness, (**c**) antibacterial characterization and in vivo infected wound healing evaluation and (**d**) wound healing mechanism. GSH: glutathione; HA: hyaluronan. Scale bars in a is 300 μm * *p* < 0.05, ** *p* < 0.01, *** *p* < 0.001. Reprinted with permission from ref. [62]. 2022, ACS.

Research has shown that CuS nanodots can be used to treat infected chronic nonhealing wounds [60,61]. The released Cu^2+^ can promote fibroblast migration and endothelial cell angiogenesis, thus accelerating wound-healing effects. In addition, CuS nanodots with photothermal effects initiate a strong antibacterial effect on drug-resistant pathogens, including methicillin-resistant *Staphylococcus aureus* (MRSA) and extended-spectrum beta-lactamase *Escherichia coli*, both in vitro and in vivo. Copper ion-incorporated hydrogel (illustrated in Figure 7) impacts the healing of infected wounds through the released copper ions, which could induce bacterial death [62].

#### 2.2.5. Magnesium Ions

Magnesium exists in the form of Mg^2+^ in humans and can serve as a cofactor in multiple enzyme catalysis reactions, participate in energy generation and transportation, coordinate protein synthesis, help transmit nerve signals, and keep muscles loose. Magnesium ions play a protective role in cardiovascular diseases, such as inhibiting calcium channels and potassium channels, inhibiting calcium ion deposition on the blood vessel wall, and creating stones. Approximately 60–80% of magnesium ions in humans are found in mitochondria, bones, myocardium, and cells.

Magnesium ions play an important role in activating coagulation FVII. However, magnesium sulfate mainly shows antithrombotic properties by inhibiting platelet aggregation and thrombus formation. In addition, magnesium ions can modulate vascular smooth muscle contraction by competing with calcium in calcium channels. Fewer magnesium ions can lead to coronary atherosclerosis or thrombosis.

Magnesium ions can promote the proliferation and migration of human umbilical vein endothelial cells and the formation of collagen and angiogenesis in skin wounds. Magnesium ions enhance the migration and adhesion of human skin fibroblasts and human immortalized keratinocytes [63,64,65]. Magnesium ions can promote Zn^2+^ into human skin fibroblasts by upregulating the expression levels of the zinc and its transporter 6/10 (ZIP6 and ZIP10) genes, enhancing signal transducer and activator of transcription 3 (STAT3) phosphorylation to induce human skin fibroblasts to differentiate into myofibroblasts, and accelerating the deposition of extracellular matrix, thereby promoting the wound healing of skin tissues [66].

Magnesium ions play an important role in immune cells and affect immune function. Magnesium ions are involved in immunoglobulin synthesis and complement activation, regulating macrophage phagocytic function and T lymphocyte maturation. In addition, magnesium ions are anti-inflammatory agents that have immune protection effects in eliminating excessive inflammation.

**Figure 8:**
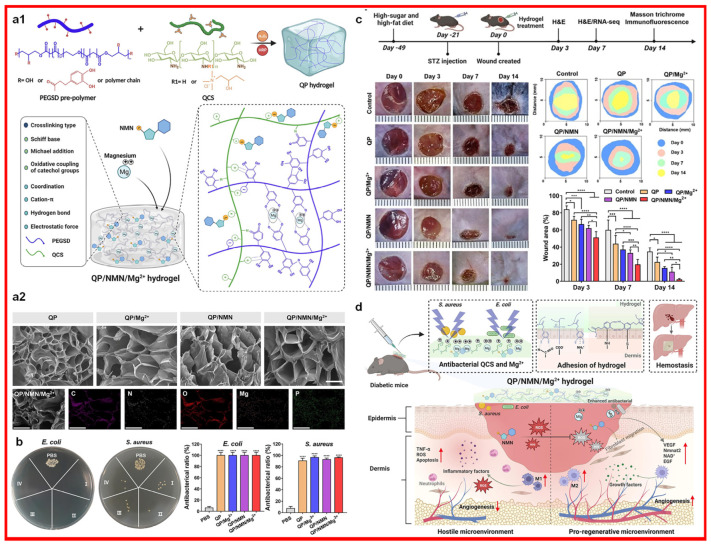
Magnesium ion incorporated hydrogel with its (**a1**,**a2**) fabrication illustration and materials characterization, (**b**) antibacterial characterization (I: QP; II: QP/Mg^2+^; III: QP/NMN; IV: QP/Mg^2+^/NMN.), (**c**) in vivo infected wound healing evaluation, and (**d**) wound healing mechanism. QP: QCS/PEGSD, quaternized chitosan/poly(glycerol sebacate)–co-poly(ethylene glycol)-g-catechol prepolymer; NMN: nicotinamide mononucleotide. Scale bars in (**a2**) contains 50 μm (top images) and 200 μm (bottom images). * *p* < 0.05, ** *p* < 0.01, *** *p* < 0.001, **** *p* < 0.0001. Reprinted with permission from ref. [69]. 2023, Elsevier.

Research has shown that magnesium ions can inhibit the production of the proinflammatory cytokines (tumor necrosis factor-α, TNF-α; interleukin 6, IL-6) in macrophages and the production of ROS and NO in immune cells, thus attenuating the neutrophil respiratory burst [67]. Magnesium ion-incorporated hydrogels are confirmed to have good proliferative capacities for fibroblasts and good inhibition effects on the NF-κB pathway (a classic transcription factor associated with inflammation and infection) [68]. Magnesium ion incorporated hydrogel (illustrated in Figure 8) could accelerate the healing of infected diabetic wounds through the released magnesium ions, which could induce bacterial death and increase the M2 macrophage count [69].

#### 2.2.6. Other Ions

In addition to the abovementioned ions, other ions, such as aluminum ions, silicon ions, and manganese ions, play less of a role in hemostatic effects. Aluminum ions combine with fibrinogen, accelerate the adhesion and activation levels of platelets, and then promote thrombosis [70]. Silicon ions can affect the adsorption of extracellular matrix components, such as collagen I, fibronectin, and vitronectin. Manganese ions can shrink local tissue [71].

## 3. Forms of Hemostatic Materials

**Figure 9:**
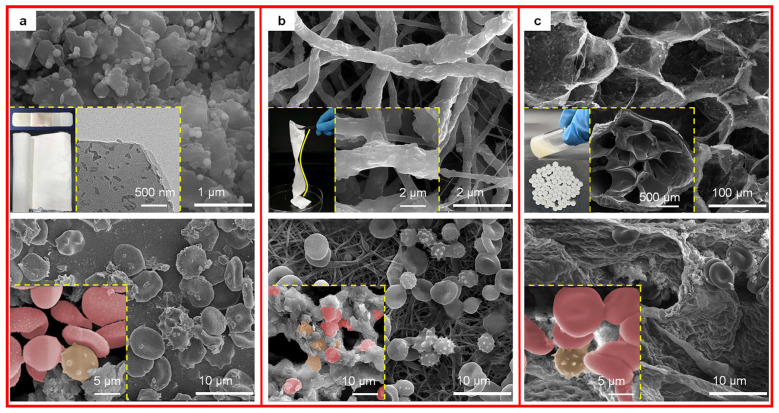
Different forms of clays based wound healing materials, such as (**a**) kaolinite based hemostatic bandage, (**b**) halloysite based hemostatic electrospun fibers, and (**c**) montmorillonite based wound healing hydrogel. Reprinted with permission from refs. [4,73]. 2023, Elsevier.

Wound healing materials with different functions have been used in different scenarios in distinct wound microenvironments, which is a realistic situation that should be considered for the use of clays in wound healing [2]. The authors conducted basic research regarding value- and effect-added mineral materials in wound healing in Figure 9, such as emerging clay composites for effective hemostasis [72], robust hemostatic bandages based on clay electrospun membranes [73], and living hydrogels with clays incorporated for wound healing. More importantly, clays can accelerate blood coagulation without introducing biohazardous effects. For example, engineered kaolinite absorbs water from the blood and concentrates the blood components at hemorrhagic sites, and more importantly, kaolin activates FXII and platelets to start the clotting cascade (blood coagulation cascade) in vivo [4,17].

### 3.1. Clay Bandages

**Figure 10:**
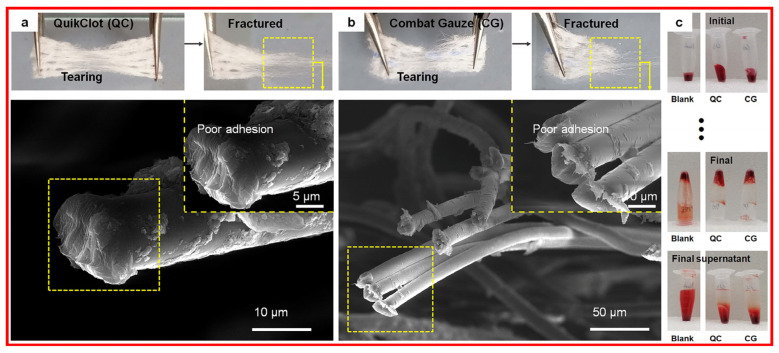
Commercial bandage with its (**a**,**b**) photos and SEM images, and (**c**) in vitro hemostatic evaluation. Reprinted with permission from ref. [74]. 2023, Elsevier.

Bandages are often used in different accident scenarios with compressible torso hemorrhage and have the advantages of being safe and efficient, with sealing anti-sticking, antimildew, and antimicrobial properties. Commercial hemostatic compounds in Figure 10 [74], including kaolinite, which is used in Combat Gauze from Z-Medica Corp.; zeolite, which is used in QuikClot from Z-Medica Corp.; and montmorillonite, which is used in WoundStat from TraumaCure Inc. Notably, a single component in bandages provides poorer hemostatic properties and requires extra functional component supplementation.

Clay-coated bandages have advantages in debridement, flexibility, wrapping, sealing, and impermeability and can prevent infection from environmental bacteria. Clays adhere to the bandage surface, preventing active component loss and residues in the wound areas [75]. Clay-coated bandages exhibit bandage characteristics and clay characteristics and can aggregate the effective components in the blood, activate platelet and coagulation cascade reactions, and accelerate the hemostasis process. In addition, the surface silanol groups from clays can enhance cell activity and result in wound healing [76].

Research has shown that clays such as montmorillonite, kaolinite, and halloysite can be combined with chitosan, polyvinyl pyrrolidone (PVP), polyethylene terephthalate (PET), and cotton through the impregnation method, resulting in a hemostatic bandage [77,78]. These bandages still have hemostatic activities after multiple rinsing treatments, and no residues remain in the wound area.

### 3.2. Clay Hydrogels

**Figure 11:**
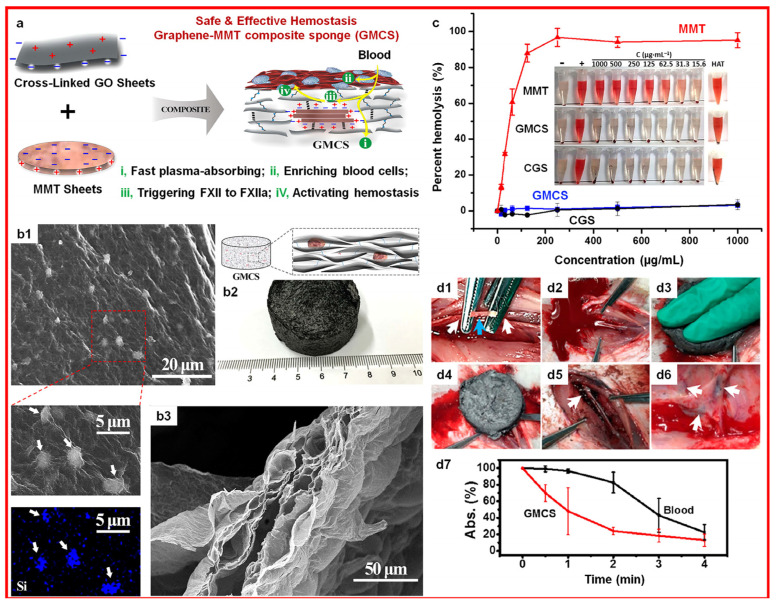
Montmorillonite-based hydrogel with its (**a**) hemostatic mechanism, (**b1**–**b3**) photo and SEM images, (**c**) hemolysis assays, and (**d1**–**d7**) hemostatic experiment in rabbits ((**d1**): separated femoral artery and transected artery; (**d2**): the wound caused hemorrhage. (**d3**): the GMCS was compressed on the wound; (**d4**): hemostasis was achieved; (**d5**): the wound was cleaned, and a clot formed (white arrow); (**d6**): the wound healing image, the white arrows denote the residue GMCS). MMT: montmorillonite; CGS: cross-linked GO sponge; GMCS: GO-MMT composite sponge. Reprinted with permission from ref. [14]. 2016, ACS.

Hydrogel materials are often used in chronic wound healing with noncompressible torso hemorrhage and have advantages such as high water content, strong tensile extension, biocompatibility, and biodegradability, thus offering a moist healing environment and bacterial isolation area. In addition, hydrogels combined with clay exhibit antibacterial functionality, hemostasis, healing, anti-inflammatory functionality, and antioxidation, and they have application prospects in the fields of biomedical engineering, such as drug delivery and tissue engineering [79]. Montmorillonite-based hydrogel (illustrated in Figure 11) could accelerate the blood coagulation through the blood or tissue fluid absorbing on the wound surface due to the hydrogel’s characteristics and the coagulation FXII chain activation with montmorillonite’s surface charges [14].

On the one hand, clays exhibit certain biological properties, such as antibacterial capabilities, healing promotion, and hemostasis, based on the characteristics of surface mixed charge, chemical stability, physiological inertness, and many active groups (hydroxyl groups). The hemostasis mechanism is based on the surface negative charge and the physical adsorption characteristics, which can directly activate platelets and endogenous coagulation FXII, effectively aggregate the main blood components, and form a blood clot around the clay without causing abnormal coagulation function [13]. The wound healing mechanism is based on the stiffness characteristics of the material and its environment, activating cell membrane surface receptors and ion channels through physical stimulation, regulating intracellular signal transmission at the transcriptomic level, affecting the expression levels of molecules related to the biological effects of migration and adhesion, and guiding correct cell migration. The antibacterial mechanism is based on the two-dimensional sheet structures and the surface active groups, which adsorb, fix, or inhibit various viruses, bacteria, and mycotoxins containing polar groups and hinder the exchange of substances between attached bacteria, bacterial communities, and the surrounding environment.

On the other hand, clay hydrogels can significantly improve the local microenvironments of wound tissues based on the micro-/nanostructure characteristics, physicochemical characteristics, and surface-interface properties. A highly ordered polymer network crosslinked structure provides a physical barrier and sealing properties, which can protect cells/tissues from environmental disturbances and prevent bacterial infection of wounded tissues [80]. Superior tensile and rheological properties provide cell/tissue adhesion and affinity, support the spreading and migration of cells, and adaptively adjust and achieve adhesion and sealing according to the irregular shape of the wound tissue. Excellent water-rich properties and the three-dimensional pore structure provide a physiological environment that highly simulates cell culture/tissue regeneration, supports cell aggregation, proliferation, and migration, prevents tissue dehydration, and ensures air permeability. Excellent swelling performance and high permeability can accommodate a large amount of water or tissue fluid. The sample swells but does not dissolve in a physiological environment, which helps to promote the absorption of wound tissue permeate. The roughness and silanol-rich characteristics of the surface/interface of the micro/nanostructure can expand the contact area between the material and cells and promote the adhesion and proliferation of cells. The stable mechanical strength and rigid building blocks provide biophysical and biochemical signals capable of guiding cell proliferation and migration at micro/nanostructured surfaces/interfaces.

Research has shown that clays, including kaolinite, halloysite, and montmorillonite loaded with nanoparticles (Fe_2_O_3_, ZnO, and Au), have been used as the core components of hydrogels for wound healing [81]. Mineral hydrogels with various functions, including antibacterial functionality, healing promotion, and hemostasis, are constructed through controllable hydrogel preparation technologies, such as in situ radical polymerization and molecular self-assembly.

### 3.3. Clay Electrospun Fibers

**Figure 12:**
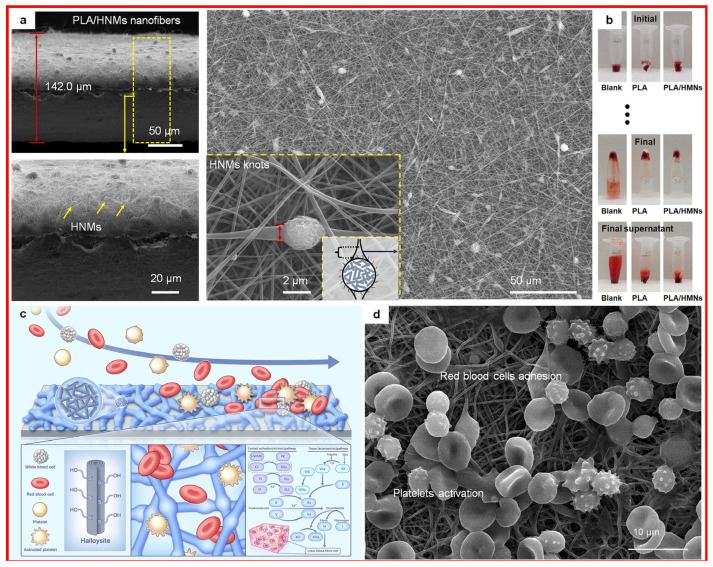
Halloysite-based electrospun fibers with its (**a**) SEM images, (**b**) in vitro hemostatic evaluation, (**c**) hemostatic mechanism, and (**d**) the blood components-electrospun fibers interaction. PLA: polyactic acid; HNMs: halloysite nanotube microspheres. Reprinted with permission from ref. [74]. 2023, Elsevier.

Electrospinning technology can continuously, stably, and economically prepare a clay-based nanofiber membrane with controllable nanofiber diameter, uniform morphology and structure, and multimaterial and multicomponent blending. In addition, the technology creates samples with high mechanical strength, a large specific surface area, and high porosity and biodegradability characteristics. Due to their high porosity, large surface area, and remodification ability, electrospun nanofibers can effectively solve the problem of powder particles falling off, compensate for the poor comprehensive performance of a single component and limited hemostatic indications, and greatly enhance the macroscopic material physicochemical structures and wound hemostatic properties. Halloysite-based electrospun fibers (illustrated in Figure 12) could accelerate blood coagulation through the blood components enrichment due to the fiber’s characteristics and the intrinsic coagulation cascade activation with the halloysite’s exposed surface charges [74].

On the one hand, the natural and diverse structure of clay can effectively alter the nanofiber morphology, obtain different nanofiber diameters and porosities of spun films, control the swelling effects and specific surface areas of spun films, and realize customized nanofiber structures. The incorporation of clays into the nanofibers can enhance their mechanical properties and play a supporting role in wound hemostasis and cell proliferation [82]. Clays can be partially exposed or attached to the nanofiber surface to form a rough texture or a unique fiber structure, such as a spindle-like structure [83]; this phenomenon increases the specific surface area and the number of active sites of the nanofiber, which can improve the ability of the fiber material to capture blood cells and to activate coagulation factors to promote the coagulation process, thereby promoting wound healing.

On the other hand, clay-based nanofiber membranes have various properties, including biocompatibility, degradation, absorption, synergistic hemostasis, synergistic coagulation, other biological properties, sealing, anti-seepage, tensile rebound, waterproofness, moisture permeability, environmental service, and other physical properties. Based on the high porosity, the large specific surface area of the fiber structure, and the similarity to natural fibrin fibers, the composite fibers can effectively capture red blood cells, platelets, etc., and achieve rapid hemostasis [84]. The hydrophilic properties of clay fibers are beneficial for absorbing water in the blood to accelerate local blood coagulation to form blood clots, enhance the aggregation, proliferation, and migration of cells on the material, and promote wound healing [85]. The abundant hemostatic active sites on the fiber and its excellent adsorption properties form a synergistic effect, which can effectively improve the hemostatic performance of the material. Excellent toughness, mechanical properties, and thermal stability can effectively fit the wound to form a seal and provide a certain compression force to meet the practical application requirements of wound dressings. The porous and breathable fiber structure can avoid the formation of an anaerobic environment in the wound, reduce the growth of anaerobic bacteria, and maintain a normal environment for wound healing.

According to the literature, clays, including kaolinite and halloysite, can be combined with PVP, polycaprolactone (PCL), polyurethane (PU), and other high molecular-weight polymers through electrospinning technology, leading to the formation of rough, fluffy nanofiber spinning films [73,86]. The composite fiber film has the characteristics of abundant hemostatic functional sites, a strong skeleton structure, and a hydrophilic surface, enabling it to rapidly aggregate blood components, activate platelets, and trigger the intrinsic coagulation pathway to promote blood coagulation. The film has excellent comprehensive performance in terms of hemostasis time, hemostatic effect, and blood loss. The hemostatic effect can meet the standard of care for commercial products.

### 3.4. Toxicities and Limitations

Those toxicities evaluations about wound healing materials should be carried out following those clinical medical orientations, which are different from oral usage, intravenous usage, external usage, etc., and that enable those standardized evaluation activities to be carried out in the micro/nano-biological studies. The evaluation of toxicities is related to influencing factors about mineral-based wound healing materials in literature [87,88,89,90,91], including mineral size distribution, mineral trace elements, human skin-associated cell lines, micro/nano-biological interactions, etc. Besides, some side effects were correlated with the excess dosages and silica sand. The mineral size distribution range from 1 nm to 1000 nm might have uncertain effects on micro/nano-biological interactions. These mineral trace elements, including iron, calcium, manganese, titanium, zinc, etc., could cause side effects in clinical medical studies, such as the excess calcium or zinc intake could induce diseases due to broken ion homeostasis, the excess Fe intake could induce diseases due to reactive -OH radicals. Mineral-based hemostatic materials have some shortcomings, such as being hard to remove from wound sites, inducing inflammation and thrombosis due to wound contamination and inefficient micro/nano-biological interactions, and therefore limiting their commercial use [91]. Nevertheless, the limitations of wound healing materials should be determined through that actual scenario, such as compressible hemostasis or incompressible hemostasis, acute or chronic wound healing, diabetic wound healing, infected wound healing, etc. Fundamental research on mineral-based wound healing materials was deficient due to their lower market share than cellulose, starch, collagen, chitosan, fibrinogen, etc.

## 4. Conclusions and Outlook

Clays are used in biomedical fields, such as antibacterial, hemostasis, and wound healing, and have received international attention. Basic exploration and technical research related to the fine processing and functional manufacturing of clays have basically eliminated the need for empirical identification techniques, such as sources, characteristics, physical clay resources, and chemical clay resources, and they have greatly reduced the dependence of clay resources on types, reserves, and grades. The following issues include the technologies regarding value- and effect-added medicinal resources, the evaluation methods regarding bio-safe medicinal materials, the wound healing mechanism at different levels, and wound dressings with different active components and multiple forms, which is something that needs to be studied in the future.

## Data Availability

The original contributions presented in the study are included in the article, further inquiries can be directed to the corresponding authors.
